# Multimodality treatment approach in a patient with EGFR-mutated NSCLC and leptomeningeal metastases: A case report and literature review

**DOI:** 10.1097/MD.0000000000043436

**Published:** 2025-07-18

**Authors:** Long-Hai Shen, Xin-Xin Yan

**Affiliations:** a Department of Oncology, Panjin Liaohe Oilfield Gem Flower Hospital, Panjin, Liaoning Province, China; b Department of Geriatrics I, Aerospace Center Hospital, Peking University Aerospace School of Clinical Medicine, Beijing, China.

**Keywords:** intrathecal perfusion, leptomeningeal metastases, non-small cell lung cancer, pemetrexed disodium

## Abstract

**Rationale:**

Leptomeningeal metastases in pulmonary adenocarcinoma patients carrying epidermal growth factor receptor mutations present a critical therapeutic dilemma. However, there is no established standard therapy following drug resistance. Novel treatment modalities are urgently needed.

**Patient concerns:**

We report a 62-year-old nonsmoking man who presented with multiple bilateral pulmonary nodules and underwent left lower lobectomy in October 2012.

**Diagnosis:**

Pathological examination confirmed poorly differentiated adenocarcinoma with neuroendocrine differentiation, classified as clinical stage IV.

**Interventions:**

The patient was initially treated with oral osimertinib; however, resistance developed after 6 months. Genetic analysis revealed an epidermal growth factor receptor exon 21 mutation. After a comprehensive treatment plan that included targeted therapy, radiotherapy, chemotherapy, and concurrent therapies. The patient achieved a total survival time of 28 months following the diagnosis of leptomeningeal metastases.

**Outcomes:**

The multimodality treatment method described in this case, incorporating whole-brain radiotherapy, intrathecal chemotherapy (dose escalation), and concurrent therapies, prolonged the patient’s survival.

**Lessons:**

Integrating a multimodal treatment plan that includes whole-brain radiotherapy and intrathecal administration of pemetrexed disodium could offer a promising treatment option for lung cancer patients with leptomeningeal metastasis.

## 1. Introduction

Pulmonary adenocarcinoma is the leading subtype of lung, with an increasing incidence of 40% to 60%. Leptomeningeal metastases (LM) occurred in approximately 5% to 8% of patients with advanced lung cancer, with a poor prognosis.^[1]^ The median survival of patients with LM is about 3 months.^[1]^ Meninges were distributed in the brain and spinal cord, cancer cells can spread to the meninges through the blood or the cerebrospinal fluid.^[2]^ Non-small cell lung cancer (NSCLC) patients with epidermal growth factor receptor (EGFR) mutations had a higher frequency of brain metastases.^[3]^ Mesenchymal-epithelial transition factor (C-MET) amplification has been found in NSCLC, and predicts both resistance to EGFR-tyrosine kinase inhibitors (TKIs) and poorer survival.^[4]^ Treatment options for patients with recurrent meningeal metastases are limited. Due to the connection between the brain and spinal cord, whole-brain radiotherapy (WBRT) alone is not effective in treating LM. At present, oral osimertinib was the main method of treatment for LM, with a median progression-free survival (PFS) time of 8.6 months.^[5]^ Once drug resistance occurred, treatment options include craniospinal radiotherapy, intrathecal chemotherapy and systemic chemotherapy. The use of intrathecal chemotherapy can be limited by inadequate central nervous system penetration. Hematologic tumor targeted drugs such as methotrexate and cytarabine were often selected as intrathecal chemotherapeutic drugs, which had few treatment options and unclear curative effects. Additionally, these intrathecal drugs is often poorly tolerated due to associated neurotoxicity. However, intrathecal infusion chemotherapy with pemetrexed was safe and effective in treating meningeal metastases from lung cancer. Here we present 1 case of EGFR-mutated pulmonary adenocarcinoma and LM with prolonged survival after administration of a treatment combining concurrent or sequential EGFR-TKI, radiotherapy and intrathecal chemotherapy, and provide a review of the literature.

## 2. Case presentation

**Figure 1.**
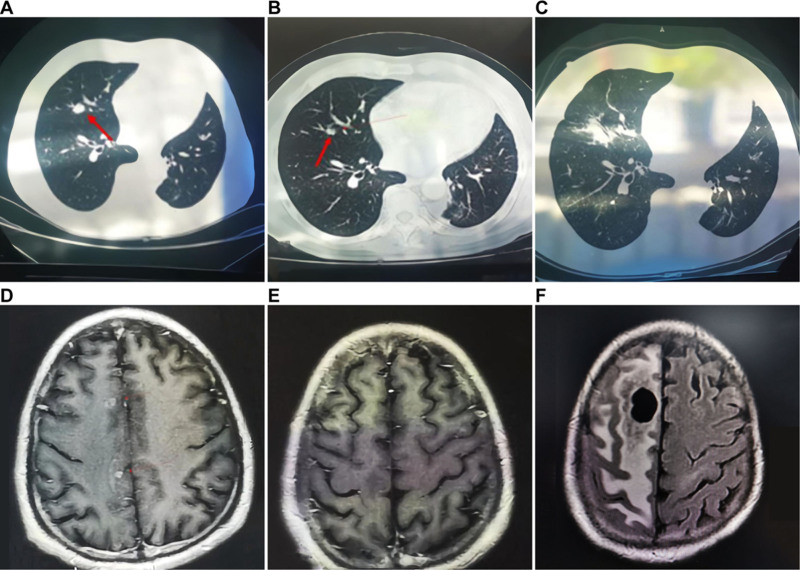
(A) Positive finding by CT scan before chemotherapy: nodules in the right upper lung lobe. (B) Positive finding by CT scan after chemotherapy: nodules in the right upper lung lobe. (C) CT scan after radiotherapy shows complete remission of lung nodules was found after 1.5 mo (D) Head MRI showed 2 brain metastatic lesions with 1 cm in diameter (E) Clinical response after gamma knife radiotherapy by head MR showed no brain metastasis. (F) Head MRI showed giant metastasis around the OMMAYA sac. CT = computerized tomography, MRI = magnetic resonance imaging.

A 62-year-old male with no smoking history underwent left inferior lobectomy in October 2012 at China Medical University Hospital due to multiple bilateral pulmonary nodules. The patient was previously in good health. Pathological examination confirmed poorly differentiated adenocarcinoma with neuroendocrine differentiation, classified as clinical stage IV. The patient received 4 cycles of chemotherapy consisting of paclitaxel (240 mg on Day 1) and cisplatin (40 mg on Days 1–3) every 3 weeks. By January 2014, the right upper lobe nodules progressed to approximately 1.8 cm in size (Fig. 1A). Subsequently, he received 6 cycles of chemotherapy with Gemcitabine (1.6 g on Days 1 and 8) and cisplatin (40 mg on Days 1–3), achieving partial response and stable disease (SD) (Fig. 1B).

In January 2016, the right upper lobe nodules progressed again. The patient underwent local conformal radiotherapy (66 Gy in 33 fractions), resulting in complete remission of lung nodules after 1.5 months (Fig. 1C). The patient experienced no adverse effects from radiotherapy, and the PFS was10 months. MRI of the head revealed 2 brain metastatic lesions (1 cm in diameter) (Fig. 1D), and bone ECT detected multiple bone metastases. Intracranial gamma knife radiotherapy led to complete remission of the intracranial lesions after 1 month (Fig. 1E). The patient received 4 cycles of chemotherapy with gemcitabine (1.6 g on Days 1 and 8) and cisplatin (40 mg on Days 1–3).

In April 2017, the patient presented with sudden headache, projectile vomiting, limb numbness, and stiff neck, which progressively worsened. Brain magnetic resonance imaging (MRI) showed no abnormalities, and mannitol was ineffective in reducing cranial pressure. Lumbar puncture revealed light-yellow cerebrospinal fluid (CSF), with carcinoma embryonic antigen (CEA) levels of 10 ng/mL in plasma and 42 ng/mL in CSF, leading to a diagnosis of pulmonary adenocarcinoma with meningeal metastases. The first-line treatment consisted of weekly intrathecal injections of methotrexate (10 mg) and dexamethasone (5 mg). However, symptoms worsened after 2 weeks, and the patient’s performance status declined to 3. Next-generation sequencing (NGS) revealed an EGFR exon 21 mutation (L858R). The patient’s condition improved with second-line treatment of oral osimertinib (160 mg QD), with performance status improving from 3 to 1 within 1 week. Resistance to osimertinib developed after 6 months, with NGS indicating concurrent C-MET amplification. The third-line treatment involved a combination of osimertinib (160 mg QD) and cabozantinib (50 mg QD), which stabilized the disease for 3 months.

In January 2018, the patient developed tinnitus, deafness, ataxia, drooping eyelids, and right exotropia, indicating disease progression. NGS analysis identified mutations in EGFR, neuroblastoma rat sarcoma gene viral oncogene homolog (N-RAS), and phosphatidylinositol-4, 5-bisphosphate 3-kinase catalytic subunit alpha (PIK3CA). Following whole-brain radiotherapy (WBRT) (30 Gy in 10 fractions), the blood-CSF barrier was opened, allowing for 8 cycles of chemotherapy with bevacizumab (500 mg on Day 1) and pemetrexed (800 mg on Day 2) every 3 weeks, leading to symptom improvement for 9 months.

In October 2018, the patient experienced dizziness and lower limb inflexibility, with CEA levels rising to 40 ng/mL in blood and 480 ng/mL in CSF. Given the restoration of the blood–brain barrier (BBB), the fifth-line treatment involved intrathecal infusion of pemetrexed (50 mg on Days 1 and 4) 3 times a week. Although initial CSF analysis showed elevated CEA levels (440 ng/mL), symptoms improved after 15 days of intrathecal pemetrexed. The treatment continued until January 2019 when symptoms worsened again, indicating disease progression. NGS reexamination showed persistent EGFR L858R mutation, new proto-oncogene tyrosine-protein kinase receptor (RET) mutations, and disappearance of previous N-RAS mutations. The sixth-line treatment involved osimertinib combined with cabozantinib, stabilizing the disease for 1 month.

**Figure 2.**
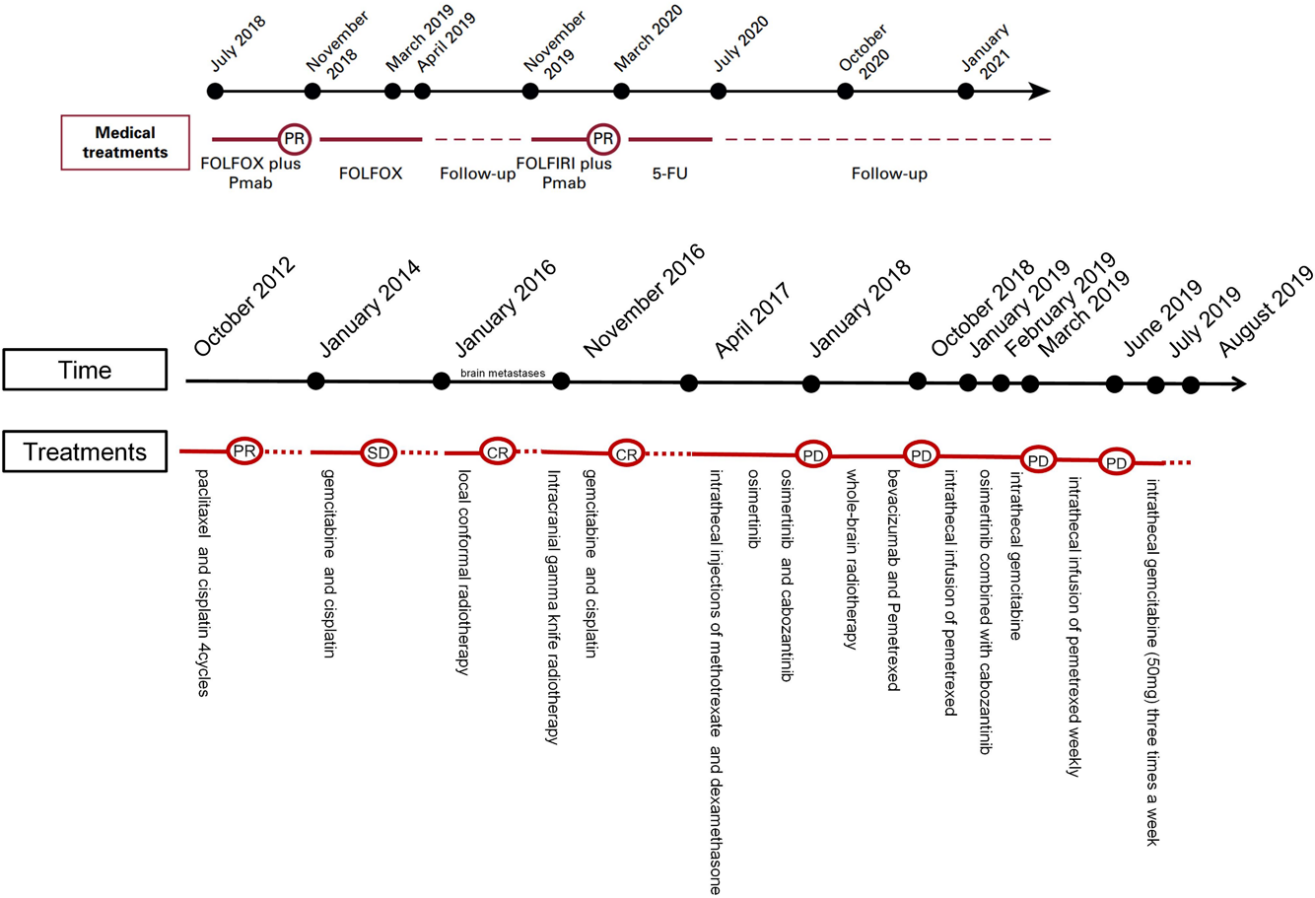
The medical treatments, time of treatment, and treatment outcomes of this patient.

In February 2019, the patient underwent Ommaya sac implantation. Following surgery, he received intrathecal gemcitabine (10 mg) 3 times a week, leading to slight symptom improvement. However, disease progression continued in March 2019, with an increased gemcitabine dose to 40 mg proving ineffective. The patient ultimately lost lower limb function, with performance status declining to Grade 3. The eighth-line treatment with intrathecal Pemetrexed (50 mg) weekly achieved SD for 3 months. By June 2019, symptoms worsened again, and the ninth-line treatment involved intrathecal gemcitabine (50 mg) 3 times a week, resulting in 1 month of SD. In July 2019, the patient experienced consciousness disturbances and left lower limb pain. MRI revealed significant metastasis around the Ommaya sac with extensive cerebral edema (Fig. 1F). Unfortunately, the patient succumbed to cerebral herniation in August 2019. The medical treatments, time of treatment, and treatment outcomes of this patient are shown in Figure 2.

## 3. Discussion

This research highlights a case of advanced lung adenocarcinoma accompanied by meningeal metastasis, characterized by the presence of the EGFR L858R mutation. Following the diagnosis of leptomeningeal metastasis (LM), the patient exhibited an overall survival (OS) of approximately 28 months. The administration of osimertinib proved to be an effective intervention for managing LM associated with the EGFR L858R mutation. The PFS achieved during second-line treatment with osimertinib was recorded at 6.0 months. Notably, the most effective therapeutic strategy involved a combination of concurrent whole-brain radiotherapy (WBRT) and intrathecal pemetrexed chemotherapy, which resulted in a clinical improvement lasting for 3 months. Additionally, through the escalated dosing of gemcitabine in conjunction with pemetrexed for seventh to ninth-line treatment, the patient’s disease remained controlled for a duration of 6 months in the terminal phase of care.

Treatment of pulmonary adenocarcinoma with meningeal metastasis remains a significant challenge. Patients have a poor prognosis, and there are currently no standard treatments. WBRT has been the most widely used treatment for brain metastases patients, but the treatment efficacy is often short-lived. The QUARTZ trial suggests that WBRT provides no benefit compared with best supportive care for patients with poor-prognosis NSCLC with asymptomatic brain metastases.^[6]^ Following radiotherapy, no standard treatments have been recommended. Alternative therapies encompass both traditional chemotherapy and systemic chemotherapy. Traditional chemotherapy has a limited role because of the presence of BBB, usually applied to patients with relapsed disease after WBRT.^[7]^ It has been suggested that meningeal metastatic tumors can disrupt the BBB^[8]^ and decrease P-glycoprotein expression, leading to increased permeability of BBB and higher response to chemotherapy.^[9]^ Currently, there are limited reports about the use of systemic chemotherapy for LM. Treatment efficacy has been observed in a limited number of regimens that have adequate BBB penetration. Recently, system chemotherapy such as EGFR-TKIs with better BBB penetration have been found increasingly used.^[10,11]^ Intrathecal chemotherapy is commonly used in the patients with cancers metastasis to the CNS.^[12]^ However, the efficacy is not well established, and it is often associated with significant neurotoxicity. With the complexities of treatment options, a comprehensive multimodality approach is needed to optimize treatment selection. In our study, combining whole-brain radiotherapy with bevacizumab and pemetrexed significantly extended PFS for 9 month in our case.

Rapid infusion of high-dose intravenous methotrexate (HD-MTX) was demonstrated to penetrate the BBB and provide durable remissions for LM in lung cancer.^[13]^ Intrathecal methotrexate is widely used in the patients with cancers metastasis to the CNS.^[12]^ Therefore, integrating HD-MTX into a multimodality treatment plan was suggested as a good choice for therapy.^[14]^ Dexamethasone is a powerful steroid medication that offers significant relief by alleviating headaches and improving neurological symptoms.^[15]^ In this case, methotrexate and dexamethasone were given as first-line treatment, but no improvement was found. Lung cancer patients with EGFR mutations have high prevalence of brain metastases. The patient experienced no significant adverse effects from the TKI medication.^[16,17]^ EGFR mutations in LM are an important predictive factor for first-line, or second-line treatment with EGFR-TKIs therapy.^[18]^ The patient was detected EGFR mutant, and the second-line treatment used the third-generation EGFR-TKI osimertinib (160 mg once a day), and the progression-free time was 6 months. Genomic analysis also showed C-Met amplification for this patient, and osimertinib resistance occurred after 6 months. C‐Met amplification is a mechanism of acquired resistance to EGFR‐TKIs, which was reported in about 20% of NSCLC cases following EGFR‐TKI treatment.^[19]^ Patients with c-MET amplification may derive clinical benefits from c-MET inhibitor cabozantinib^.[20]^ The combination of 2 TKIs, cabozantinib and osimertinib was used as third-line treatment, and SD was obtained for 3 months.

In this case, bevacizumab plus pemetrexed were applied in forth-line treatment, concomitant with WBRT. Reports on this combination therapy are limited, but the potential is promising. Bevacizumab and pemetrexed, 2 effective and well-tolerated agents, have been highly recommended as maintenance therapies for patients with advanced NSCLC.^[21–25]^ The combination maintenance regimen of bevacizumab and pemetrexed have indicated clinical benefit of significantly improved PFS and intracranial PFS versus single treatment alone in patients with advanced adenocarcinoma and brain metastases.^[26]^ Chemotherapy drugs can enter the brain tissue when radiation destroys the BBB. Additionally, chemical drugs have the potential to make brain tumor cells more sensitive to radiotherapy. Since WBRT alone was not effective in treating meningeal metastases, in order to increase the efficacy, incorporating WBRT and intrathecal chemotherapy or EGFR-TKIs could further improve the treatment outcome. In this study, the concurrent and sequential combination treatment modalities were applied. Application of bevacizumab-assisted pemetrexed after WBRT opened the blood-cerebrospinal fluid barrier achieved a SD response up to 9 months. On the other hand, considering that the blood-cerebrospinal fluid barrier may self-repair after radiotherapy, intrathecal infusion of pemetrexed may be still effective. Following treatment with cabozantinib and osimertinib, the tumor progressed within a month, likely due to drug resistance. Nevertheless, clinical studies indicate that combining chemotherapy with whole-brain radiation therapy (WBRT) provides considerable benefits for NSCLC patients with brain metastases, compared to WBRT alone.^[27]^ However, other reports showed controversial results, and suggested that chemotherapy concurrent with WBRT did not show efficacy on NSCLC patients with brain metastases and increases the incidence of adverse events.^[28]^ Another study by Pan et al, concluded that a combination of intrathecal chemotherapy and radiation therapy is an optimal treatment option for leptomeningeal metastasis in metastatic solid tumors. However, this treatment approach is associated with significant neurotoxicity and does not show a significant OS and clinical response advantage.^[29]^ There were no significant neurotoxic side effects in our case. Additionally, intrathecal chemotherapy administration requires frequent lumbar punctures, which adds additional procedure related risks. In this study, the patient underwent OMMAYA sac implantation in neurosurgery before the seventh-line treatment of gemcitabine in order to reduce the number of lumbar punctures. Both pemetrexed and gemcitabine are chemotherapeutic agent that inhibits DNA synthesis in dividing cancer cells. Reports have shown that gemcitabine combinations with other agents exhibit synergistic effects and produce important survival advantages in advanced NSCLC^.[30,31]^ Postoperative intrathecal injection of increased doses of gemcitabine was effective. Considering the circulating fluidity of the cerebrospinal fluid and the active excretion of P-g glycoprotein, the duration of action of chemotherapy drugs in the cerebrospinal fluid may be relatively short. Application of increased doses or increased frequency of intrathecal infusion chemotherapy may allow the original drug-resistant intrathecal chemotherapy regimen to continue to be effective. Pan et al reported that the main adverse effects of intrathecal pemetrexed chemotherapy were bone marrow suppression, liver damage and radicular neuritis. In our case, no significant adverse effects occurred with the increased frequency of dosing.^[29]^ However, in this study, the patient had no adverse reactions with intrathecal injection of 50 mg of pemetrexed and gemcitabine. Other preliminary evidence also suggests that courses of intravesical gemcitabine are safe and as effective or more effective than other chemotherapeutic agents for cancer recurrence. Although the OMMAYA sac can reduce the operation of lumbar puncture, but it can cause intracranial implantation metastasis. In this patient, multiline treatment of meningeal metastasis was effective, and the condition of parenchymal metastasis was ignored.

During the treatment period, different treatment modality and their combination, different drug dosing and timing of application were explored. The patient had an overall survival of about 28 months after diagnosis of LM, which proved good treatment effect compared with many other survival data reported in the literature. We also learned from this case that the combination therapy with bevacizumab and pemetrexed is effective. The best treatment modality was the concurrent WBRT and intrathecal chemotherapy with bevacizumab and pemetrexed, the patient had good clinical benefit with a symptomatic improvement of about 9 months. This study also presents an advanced lung cancer case with meningeal metastasis harboring EGFR exon 21 L858R mutation, which was effectively treated using third-generation TKI osimertinib. The PFS for the second-line treatment was 6.0 months, which might be related to the sensitivity to EGFR gene mutations. The cerebrospinal fluid CEA of patient began to decrease 3 days after the first intrathecal chemotherapy, and his symptoms began to improve after 2 weeks, indicating that the detection of cerebrospinal fluid markers may determine the change of the condition earlier than the clinical symptoms.

Co-existence of EGFR and c-MET mutations was seldom reported, only accounting for about 5% of EGFR-mutated patients. And in this case, the role of the detected mutations of N-RAS, PIK3CA and RET genes on the limited PFS and OS remains to be elucidated. Future studies with a large sample should be carried out to confirm the efficiency and clarify underlying mechanisms. Genomic analysis may be helpful to identify patients most likely to response to this treatment regimen.

## 4. Conclusion

In conclusion, the multimodality treatment method described in this case, incorporating whole-brain radiotherapy, intrathecal chemotherapy and systemic chemotherapy, prolonged the patient’s survival. It may provide a good experience and reference for further study of optimized therapies for lung cancer meningeal metastasis.

## Author contributions

**Data curation:** Long-Hai Shen.

**Formal analysis:** Long-Hai Shen.

**Funding acquisition:** Xin-Xin Yan.

**Project administration:** Long-Hai Shen.

**Resources:** Long-Hai Shen.

**Software:** Long-Hai Shen.

**Supervision:** Long-Hai Shen.

**Validation:** Long-Hai Shen.

**Visualization:** Long-Hai Shen.

**Writing – original draft:** Long-Hai Shen.

**Writing – review & editing:** Xin-Xin Yan.
